# Productivity Improvement of Human Papillomavirus-like Particles in Insect Cells Using Hyper-Expression Baculovirus Vector

**DOI:** 10.3390/vaccines13101006

**Published:** 2025-09-25

**Authors:** Jae-Bang Choi, Ji-Hoon Lee, Eun-Ha Kim, Jae-Deog Kim, Seong-Yeong Kim, Jong-Min Oh, Soo-Dong Woo, Hyunil Kim, Beom-Ku Han

**Affiliations:** 1 Optipharm Inc., Osong 28158, Republic of Korea; leejih@optipharm.co.kr (J.-H.L.); keunha@optipharm.co.kr (E.-H.K.); kimjd@optipharm.co.kr (J.-D.K.); ksyoung@optipharm.co.kr (S.-Y.K.); ojm@optipharm.co.kr (J.-M.O.); hikim@optipharm.co.kr (H.K.); bkhan@optipharm.co.kr (B.-K.H.); 2 Department of Agricultural Biology, College of Agriculture, Life & Environment Science, Chungbuk National University, Cheongju 28644, Republic of Korea; sdwoo@cbnu.ac.kr

**Keywords:** HPV, VLP, L1 protein, hyper-expression baculovirus vector system, vaccine

## Abstract

**Background/Objectives**: Virus-like particle (VLP) vaccines based on human papillomavirus (HPV) L1 proteins have high efficacy for preventing cervical cancer and other HPV-associated diseases. The production yields of commercial HPV VLPs remain suboptimal. We aimed to improve HPV VLP production efficiency using a hyper-expression vector system for the expression of L1 proteins of four major HPV serotypes—HPV 6, 11, 16, and 18. **Methods**: HPV L1 proteins were expressed in *Trichoplusia ni* (Hi5) insect cells via a hyper-expression baculovirus vector system. Following cell lysis using a microfluidizer, VLPs were purified through a two-step chromatographic process. Particle morphology was characterized using transmission electron microscopy and dynamic light scattering. Immunogenicity was evaluated using a murine model; mice received three intramuscular injections of the purified quadrivalent VLPs. The resulting IgG and neutralizing antibody responses were compared with those elicited by the commercial quadrivalent vaccine, Gardasil. **Results**: The L1 proteins from HPV 6, 11, 16, and 18 were successfully expressed at high levels in Hi5 cells, forming uniformly sized VLPs with hydrodynamic diameters of 50–60 nm. The average production yield of the quadrivalent VLPs exceeded 40 mg/L, an improvement over conventional yields. The candidate VLPs elicited strong HPV-specific IgG and neutralizing antibody responses in mice, comparable to those induced by Gardasil. **Conclusions**: The hyper-expression baculovirus vector system enables high-yield production of HPV L1 VLPs with desirable structural and immunogenic properties. This approach holds promise for the cost-effective and scalable manufacturing of next-generation HPV VLP vaccines, facilitating broader global access to HPV immunization.

## 1. Introduction

Cervical cancer is a prevalent cancer affecting women worldwide. According to the GLOBOCAN 2022 estimates, approximately 662,300 new cases and 348,900 deaths were recorded globally in 2022 [1,2].

Human papillomavirus (HPV) represents the most notable etiological factor of cervical cancer, with HPV DNA detected in more than 99% of cervical cancer cases [3,4]. HPV, a double-stranded DNA virus with a genome of approximately 8 kb, comprises over 100 genotypes [5,6]. High-risk HPV types are associated with an increased risk of malignant transformation and are recognized as oncogenic viruses. Among these, HPV types 16 and 18 are responsible for more than 70% of cervical cancer cases, while types 6 and 11 are implicated in approximately 90% of genital wart occurrences [7]. Natural HPV infection induces detectable antibody formation in only approximately 50–60% of women [8]. Therefore, prophylactic vaccination is essential for preventing HPV infection. Three prophylactic virus-like particle (VLP)-based HPV vaccines—Gardasil^®^, Gardasil^®^9, and Cervarix^®^—are currently licensed and widely available [9,10,11]. Additionally, several newer vaccines have received approval in recent years, including Cecolin^®^ (bivalent HPV-16/18, approved in China in 2020 and World Health Organization [WHO]-prequalified in 2021), Walrinvax^®^ (bivalent HPV-16/18, approved in China in 2022), and Cervavac^®^ (quadrivalent HPV-6/11/16/18, launched in India in 2022).

The HPV L1 protein possesses the intrinsic ability to self-assemble efficiently into capsid-like structures known as VLPs in vivo, as demonstrated in studies using recombinant baculovirus and yeast expression systems [12,13,14,15]. VLPs are self-assembling, non-infectious nanostructures that mimic the morphology of normal viruses but lack viral genetic material. The absence of a viral genome not only ensures that VLPs are non-replicative and non-infectious but also contributes to their enhanced safety profile compared to attenuated or inactivated viral vaccines [16,17]. VLPs are known to induce potent humoral and cellular immune responses due to their structural similarity to actual virions [18,19], making them a promising approach for vaccine development [20]. The baculovirus expression vector system is widely utilized for producing recombinant proteins in insect cells or larvae [21,22]. This powerful eukaryotic expression system offers several advantages for generating diverse proteins in high yields [23] and has played a crucial role in the development and production of HPV vaccines [24,25,26]. In our previous study, we developed a hyper-expression vector system utilizing a combination of baculovirus promoters and transcription factors [27]. The enhanced protein expression was initially validated using enhanced green fluorescent protein (EGFP) as a reporter protein. While acknowledging that expression efficiency can vary depending on the specific protein being studied, this research sought to assess the performance of our optimized vector system in expressing the HPV L1 protein, an antigen of notable clinical relevance. In this study, we applied our previously developed hyper-expression vector to produce the HPV L1 protein and assessed its yield and immunogenicity. By examining the expression levels, purification efficiency, and the ability of the produced L1 protein to form VLPs, we sought to validate the effectiveness of our vector system for practical applications. Furthermore, this study aimed to demonstrate the potential industrial applicability of our hyper-expression vector by providing comprehensive data supporting its potential use for large-scale production of biopharmaceuticals, particularly in the context of vaccine manufacturing.

## 2. Materials and Methods

### 2.1. Cell Lines and Culture Media

The *Spodoptera frugiperda* cell line (Gibco™ Sf9; Gibco, Grand island, NY, USA) was kept at 27 °C in SF900™ III SFM, a serum-free medium (Gibco, Grand island, NY, USA), while *Trichoplusia ni* BTI-TN5B1-4 (High Five™, Hi5) cells (Boyce Thompson Institute, Ithaca, NY, USA) were cultured in Express Five™ SFM (Gibco, Grand island, NY, USA). Routine cell cultures were performed in 125 mL or 1000 mL shake flasks with a 20% working volume at 130 rpm in an ISS-7100R incubator (JEIO TECH, Daejeon, Republic of Korea) at 27 °C; cells were subcultured every 3–4 days.

### 2.2. Construction of Hyper-Expression Vectors

The HPV L1 gene sequences for HPV types 6, 11, 16, and 18 were identified through a comprehensive GenBank search, and prototype sequences for each type were selected. To enable efficient expression in the Sf9 cell line, codon optimization was performed. To improve protein solubility, the selected L1 gene sequences were further modified by truncating the N-terminal and C-terminal regions to remove the nuclear localization signals previously reported to affect solubility [26]. The modified HPV L1 sequences were synthesized (Sequence S1), including several restriction enzyme sites required for cloning. This work was carried out by BIONICS Inc. (Seoul, Republic of Korea). All vectors were generated with In-Fusion cloning (Takara Bio USA, Inc., San Jose, CA, USA ). These constructs were built in the hyper-expression vector.

### 2.3. Generation of Recombinant Viruses

Recombinant viruses were generated with a co-transfection of Sf9 cells. This involved recombination of the hyper-expression vector plasmid phyper-HPV-L1, containing the HPV L1 sequence, with the baculovirus genome (flashback ULTRA DNA). The process was conducted according to the manufacturer’s instructions for flashBAC™ ULTRA (Oxford Expression Technologies Ltd., Oxford, Oxfordshire, UK).

### 2.4. Sodium Dodecyl Sulfate Polyacrylamide Gel Electrophoresis (SDS-PAGE) and Western Blot

Viral infection was performed using 1 × 10^8^ Hi5 cells in a 500 mL Erlenmeyer flask at a multiplicity of infection (MOI) of 1 plaque-forming unit/cell. For protein analysis, the infected cell cultures were incubated for 72 h at 27 °C with shaking at 130 rpm in an ISS-7100R incubator (JEIO TECH Co., Ltd., Seoul, Republic of Korea). Cells were harvested via centrifugation, and the resulting cell pellets were stored at −80 °C until further analysis. For cell lysis, pellets were resuspended in phosphate-buffered saline (PBS) containing 1% Triton X-100 and incubated appropriately. The lysates were then mixed with protein sample buffer and boiled for 10 min. Protein samples were separated with 12% SDS-PAGE and transferred to a polyvinylidene fluoride membrane. The membrane was blocked with 5% skim milk in Tris-buffered saline containing 0.05% Tween 20 and probed with an HPV L1 monoclonal antibody (Camvir 1; Abcam, Cambridge, Cambridgeshire, UK). After washing, the membrane was incubated with a horseradish peroxidase (HRP)-conjugated goat anti-mouse immunoglobulin G (IgG) H&L secondary antibody (Abcam, UK), and bound antibodies were detected using WESTSAVE ECL solution (AbFrontier, San Jose, CA, USA) according to the manufacturer’s instructions.

### 2.5. Production and Purification of HPV L1 VLPs

Protein expressions were carried out in Hi5 cells. HPV 6, 11, 16, and 18 serotypes L1 proteins were expressed in 200 mL of Express Five^TM^ SFM media (Gibco, Grand island, NY, USA). Briefly, Hi5 cells were seeded at a density of 1 × 10^6^ cells/mL in 200 mL of the medium in a 1 L Erlenmeyer flask (Corning Inc., Corning, NY, USA) and subsequently infected with recombinant virus. Hi5 cells were infected with recombinant baculoviruses expressing the L1 proteins of HPV types 6, 11, 16, and 18, respectively, at an MOI of 1 and incubated at 27 °C for 72 h with shaking at 130 rpm. Cells were harvested via centrifugation at 1000× *g* for 10 min and washed with PBS. Next, the cell pellets were resuspended in an equal volume of lysis buffer containing 20 mM Tris-HCl, pH 8.5, with 0.01% Tween-80. Lysis was performed using the LM20 Microfluidizer^®^ Processor (Microfluidics, Westwood, MA, USA) for one pass at 20,000 psi on ice. The lysate was clarified via centrifugation at 10,000× *g* for 20 min at 4 °C, followed by filtration through a 1000 mL bottle top vacuum filter (Corning, NY, USA). The resulting supernatant was used immediately for protein purification, which was performed using an ÄKTA go™ fast protein liquid chromatography system (Cytiva, Marlborough, MA, USA). The purification process consisted of three steps using Source 30Q resin (17127502, Cytiva, USA), CHT-II hydroxyapatite resin (HA; 157-4000, Bio-Rad, Hercules, CA, USA), and a PD-10 desalting column (17085101, Cytiva, Marlborough, MA, USA). The Source 30Q and HA resins were packed into XK26 and XK16 columns, respectively, each to a bed height of 10 cm. The PD-10 column was used in its supplied open-column configuration. The filtered lysate was supplemented with β-mercaptoethanol (BME; M6250, Sigma-Aldrich, St. Louis, MO, USA) to a final concentration of 4% (*v*/*v*) and incubated at 4 °C for 3 h to facilitate disassembly. The disassembled L1 VLP protein was then loaded onto a Source™ 30Q column (17127502, Cytiva, Marlborough, MA, USA) and eluted using a linear gradient between Source™ 30Q wash buffer (20 mM Tris-HCl, 4% BME, pH 7.5) and elution buffer (20 mM Tris-HCl, 1 M NaCl, 4% BME, pH 7.5). Each fraction was collected and analyzed with SDS-PAGE and western blotting. Fractions containing the target protein were pooled, and the sodium chloride concentration was adjusted to 1 M. Subsequently, the pooled protein was loaded onto the HA column and eluted with gradient conditions using HA wash buffer (10 mM Sodium phosphate, 4% BME, pH 6.4) and HA elution buffer (200 mM Sodium phosphate, 1 M NaCl, 4% BME, pH 6.4). The flow-through and eluted fractions were analyzed with SDS-PAGE and western blotting, respectively. Fractions containing the target protein were pooled and concentrated to 2.5 mL using the Amicon Ultra-15 centrifugal filter (10K MWCO, UFC901024, Millipore, Burlington, MA, USA). As a final step, PD-10 desalting was performed according to the manufacturer’s instructions. Briefly, 2.5 mL of the concentrated protein was loaded onto the column and eluted with 3.5 mL of reassembly buffer (0.5 M NaCl, 0.01% Tween 80 in 1× PBS). Purified protein was reassembled at 4 °C for 48 h with gentle inversion. Protein concentration was determined using the Bradford assay reagent (1863028, Thermo Fisher Scientific, Waltham, MA, USA according to the manufacturer’s protocol, and the final yield was calculated.

### 2.6. Dynamic Light Scattering (DLS) and Transmission Electron Microscopy (TEM)

To characterize the size and distribution of VLPs, DLS measurements were performed. The purified and reassembled HPV L1 VLPs were equilibrated to 25 °C, and the hydrodynamic diameter and polydispersity index of particles were determined using ZETASIZER ULTRA (ZSU5700, Malvern Panalytical, Malvern, Worcestershire, UK).

For TEM analysis, glow-discharged, carbon-coated copper grids were prepared, and negative staining was performed. HPV L1 VLP samples were adsorbed onto grids for 2 min, followed by blotting of excess buffer onto Whatman^®^ filter paper (Cytiva, Marlborough, MA, USA). Grids were then stained with Uranyless™ (Electron Microscopy Sciences, Hatfield, PA, USA)for 1 min, after which excess stain was blotted. Imaging was performed using a high-voltage EM system (JEM-1400 Plus at 120 kV and JEM-1000BEF at 1000 kV; JEOL Ltd., Tokyo, Japan) at the Korea Basic Science Institute.

### 2.7. Immunization with Purified HPV L1 VLPs

BALB/c female mice (6 weeks old) were purchased from KOATECH Inc. (Ansan-si, Gyeonggi-do, Republic of Korea) and used for the experiments. Mice were immunized thrice intramuscularly at weeks 0, 4, and 8 with HPV L1 VLPs containing 2 μg of type 6, 4 μg of type 11, 4 μg of type 16, and 2 μg of type 18, formulated with 50 μg of aluminum hydroxide gel (InvivoGen, San Diego, CA, USA) in PBS.

Positive control mice were immunized intramuscularly with Gardasil (1/10 of the human dose, i.e., 50 μL corresponded to 2 μg HPV6 VLPs, 4 μg HPV11 VLPs, 4 μg HPV16 VLPs, and 2 μg HPV18 VLPs) at weeks 0, 4, and 8. Negative control mice received PBS containing 50 μg of aluminum hydroxide gel (InvivoGen, San Diego, CA, USA). Sera were collected from the animals 2 weeks after the final immunization. All animal studies were reviewed and approved by the Institutional Animal Care and Use Committee (IACUC) of KBIO Health (Cheongju-si, Chungcheongbuk-do, Republic of Korea) for the vaccine study (approved number: KBIO-IACUC-2024-060).

### 2.8. Enzyme-Linked Immunosorbent Assay (ELISA)

IgG antibodies against HPV antigens were detected in mouse sera using a mouse anti-HPV L1 IgG ELISA kit (Alpha Diagnostic Intl. Inc., San Antonio, TX, USA) according to the manufacturer’s instructions. The kit included microtiter plates pre-coated with HPV-specific antigens. Uniformly diluted serum samples were added to each well and incubated for 1 h at 37 °C. After incubation, the wells were washed, and HRP-conjugated anti-mouse IgG was added, followed by a 30-min incubation at 37 °C. Following additional washes, 3,3′,5,5′-tetramethylbenzidine substrate solution was added, and the enzyme–substrate reaction was stopped with the addition of a stop solution. Color development was measured spectrophotometrically at 450 nm using a microplate reader (Infinite M200 Pro, Tecan Group Ltd., Männedorf, Zurich, Switzerland). The levels of HPV16 IgG antibodies in the samples were determined using optical densities according to the manufacturer’s protocol.

### 2.9. Neutralization Assay

The pseudovirus-based neutralization assay (PBNA) was performed as previously described with minor modifications [28]. HEK 293TT cells were seeded into 96-well plates at a density of 3 × 10^4^ cells per well and incubated for 6 h at 37 °C. Serum samples were serially diluted fourfold in culture medium, and each HPV PsV was added to the diluted serum. The serum–PsV mixtures were incubated at 4 °C for 1 h on a rocking shaker and then transferred to the HEK 293TT cell-seeded plates. The plates were further incubated for 72 h at 37 °C. After incubation, 100 μL of culture medium was removed from each well and replaced with 100 μL of ONE-Glo™ Luciferase Assay Reagent (Promega, Madison, WI, USA). The plates were gently shaken for 3 min to ensure thorough mixing, after which the contents were resuspended and transferred to black 96-well plates. Luminescence was measured using a GloMax^®^ microplate reader (Promega, Madison, WI, USA).

### 2.10. Statistical Analysis

Statistical significance was determined using an unpaired, two-tailed *t*-test in GraphPad Prism version 8.2.1. *p*-values were designated as follows: ns, not significant (*p* ≥ 0.05); * *p* < 0.05; ** *p* < 0.01; *** *p* < 0.001; **** *p* < 0.0001.

## 3. Results

### 3.1. Construction of Transfer Vector (pHyper-HPV-L1) and Generation of HPV-L1 Recombinant Baculoviruses

**Figure 1:**
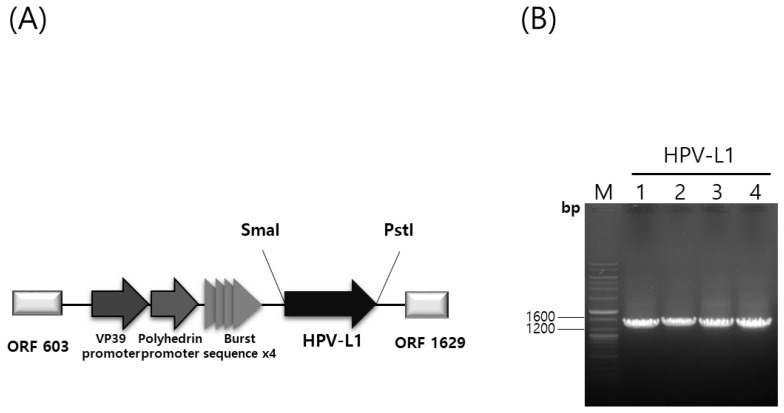
Hyper vector plasmid construction and confirmation of recombinant HPV-L1 with PCR. (**A**) Construction of a hyper-expression vector containing the HPV L1 gene. (**B**) PCR of HPV 6, 11, 16, and 18 genomes using specific L1 primers. HPV, Human Papillomavirus; PCR, polymerase chain reaction; Lane M, ladder marker; Lane 1, 2, 3, and 4, insertion result of HPV 6, 11, 16, and 18 L1 gene into recombinant baculovirus DNA amplified with specific L1 primers.

The HPV-L1 gene was cloned into the pHyper vector (Figure 1A,B). The recombinant plasmid, pHyper-HPV-L1, was confirmed with restriction enzyme digestion using *Sma*I and *Pst*I. Recombinant baculoviruses (rHPV-6-L1, rHPV-11-L1, rHPV-16-L1, and rHPV-18-L1) were generated using the flashBAC™ ULTRA system (Oxford Expression Technologies Ltd., UK). The HPV L1 gene was expressed under the control of VP39 and polyhedrin promoters with burst sequences following infection of insect cells. The presence of the HPV-L1 gene in recombinant baculoviral DNA was confirmed with polymerase chain reaction using specific primers, as shown in Figure 1B. The HPV-L1 protein of these recombinant baculoviruses was purified and propagated as described previously [29].

### 3.2. Expression and Extraction of HPV 6, 11, 16, and 18 L1 Proteins in Hi5 Cells

**Figure 2:**
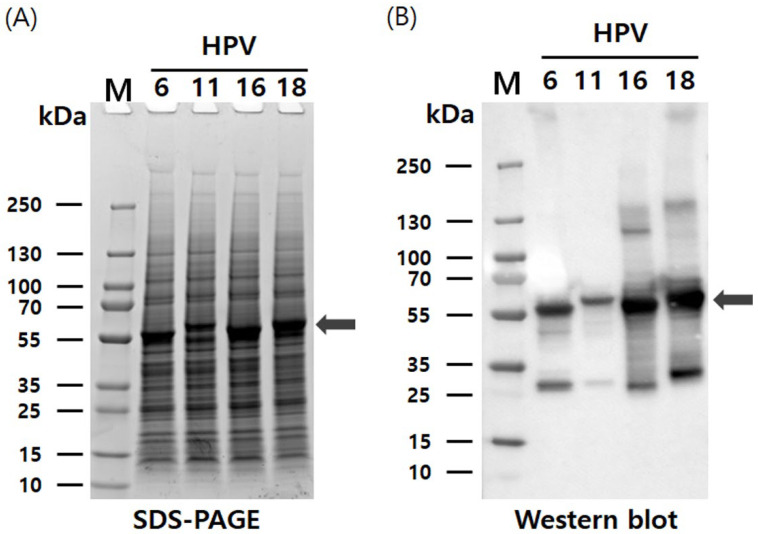
Characterization of HPV 6, 11, 16, and 18 L1 proteins using recombinant baculovirus-based pHyper vector. (**A**) Expression of HPV-L1 in Hi5 cells analyzed with SDS-PAGE. (**B**) Expression of HPV-L1 in Hi5 cells analyzed with western blotting using L1-specific antibodies. L1 protein bands are indicated by arrows. The broad-spectrum anti-HPV L1 monoclonal antibody CamVir-1 (Abcam, USA) was used to probe all L1 proteins. M, molecular weight marker; SDS-PAGE, Sodium dodecyl sulfate polyacrylamide gel electrophoresis.

**Figure 3:**
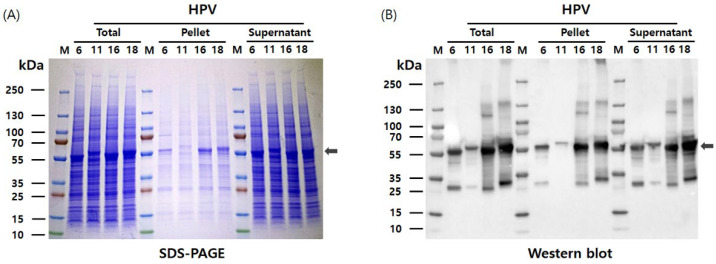
Extraction and analysis of HPV L1 protein from Hi5 cells using a microfluidizer. (**A**) SDS-PAGE analysis of soluble and insoluble fractions following microfluidizer lysis (20,000 psi, one pass), demonstrating the presence of HPV L1 proteins in the supernatant. (**B**) Western blot analysis confirming the expression and solubility of HPV L1 proteins in the corresponding fractions. The expected molecular weights of the four HPV L1 proteins are indicated by arrows.

**Figure 4:**
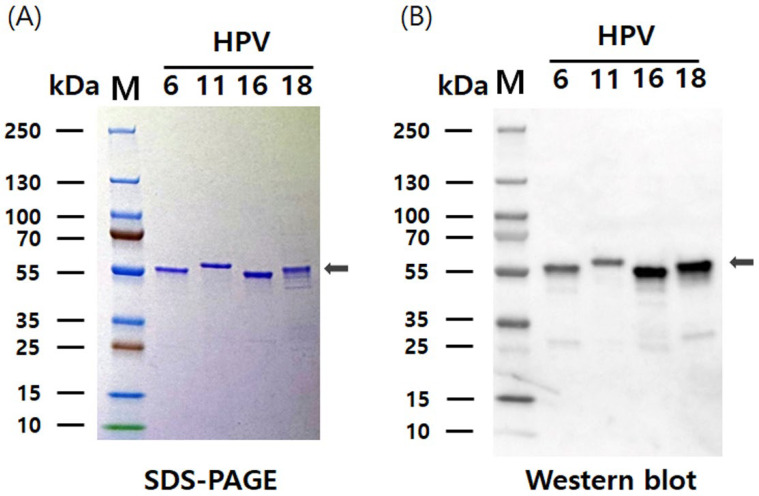
SDS-PAGE and western blot analysis of purified HPV 6, 11, 16, and 18 L1 proteins. (**A**) SDS-PAGE analysis of purified L1 proteins separated on a 12% polyacrylamide gel. (**B**) Western blot detection of the purified L1 proteins using the HPV L1-specific monoclonal antibody CamVir-1. The expected molecular weights of the four HPV L1 proteins are indicated with arrows.

The pHyper vectors were used to express the L1 proteins of four HPV types in Hi5 cells. SDS-PAGE analysis revealed that the L1 proteins were produced as the predominant band density among total cellular proteins (Figure 2). The presence of L1 proteins was further confirmed with western blotting using an anti-HPV L1 monoclonal antibody at approximately 55 kDa. Theoretical molecular weights of HPV L1 proteins for each serotype were calculated as follows: HPV6-L1 was 51.9 kDa, HPV11-L1 was 53.9 kDa, HPV16-L1 was 52.6 kDa, and HPV18-L1 was 52.7 kDa. However, the recombinant proteins expressed in insect cells using the baculovirus system exhibited higher molecular weights than predicted, likely due to post-translational modifications and other cellular processing events. These results indicate that the target proteins were highly and successfully expressed in Hi5 cells. Consistent L1 band densities were detected via SDS-PAGE across HPV types. Contrastingly, western blot analysis under identical conditions showed significant variations in band intensity (Figure 2). We attribute this divergence to the binding affinity of CamVir-1. While this monoclonal antibody targeted HPV16-L1, it exhibited broad cross-reactivity with other types. CamVir-1 antibody recognizes the FG loop region of the HPV L1 protein, and since each serotype possesses several amino acid differences within the FG loop, we predicted that the Western blot band intensity would differ depending on the serotype. As expected, when equal amounts of purified L1 protein from each serotype were quantified and analyzed, distinct differences in band intensity were observed among the serotypes (Figure 3 and Figure 4). HPV L1 protein was extracted using an LM20 Microfluidizer^®^ Processor (Microfluidics, Westwood, MA, USA). Hi5 cells infected with recombinant baculovirus were harvested 3 days post-infection, and analysis with SDS-PAGE and western blot demonstrated that a substantial amount of HPV L1 protein was present in the soluble fraction (Figure 3). Additionally, residual L1 protein was also detected in the cell pellet, suggesting that, due to high-level expression, a portion of the L1 protein formed aggregates or remained insoluble because of improper folding during expression. Although hyper-expression vectors are commonly employed to maximize target protein production, these results suggest that the choice of expression vector should be carefully optimized according to the properties of the protein being expressed.

### 3.3. Purification and Reassembly of HPV L1 VLPs

HPV L1 VLPs were purified from Hi5 cells using a protocol adapted from a previously described method for purifying HPV L1 VLPs expressed in insect cells [30]. The purification process consisted of two-step chromatography and filtration, resulting in the isolation of the HPV L1 protein in pentameric form. Subsequently, the purified L1 protein was assembled into VLPs that closely resembled the structure of native HPV virions. Assembly of HPV VLPs was achieved with buffer exchange into a high-salt solution (1 M NaCl) at neutral pH (7.2) and the removal of reducing agents. SDS-PAGE and western blot analyses demonstrated that the purity of HPV 6, 11, 16, and 18 L1 VLPs after the two-step chromatography exceeded 90% (Figure 4). The yield of L1 protein VLPs varied depending on the serotype, and optimization of culture conditions resulted in production levels of 67 mg/L for HPV6 L1, 51 mg/L for HPV11 L1, 25 mg/L for HPV16 L1, and 16 mg/L for HPV18 L1. The average production yield of the quadrivalent HPV L1 VLPs reached approximately 40 mg/L. These yields are substantially higher than previously reported values of approximately 10 mg/L for conventional L1 VLPs, excluding chimeric VLPs [31,32]. These results are consistent with previous studies using hyper-expression vectors with EGFP, demonstrating enhanced expression and yield of the target protein. Therefore, this study further supports the commercial potential of hyper-expression vectors for large-scale protein production.

### 3.4. Characterization of Purified HPV 6, 11, 16, and 18 VLPs

**Figure 5:**
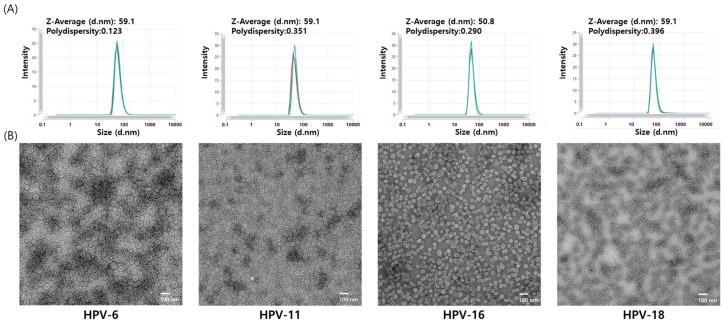
Dynamic light scattering (DLS) and transmission electron microscopy (TEM) analysis of HPV 6, 11, 16, and 18 VLPs. (**A**) Hydrodynamic size distribution of purified HPV VLPs measured with DLS. DLS measurements were performed in triplicate, and the analytical results from each replicate are shown individually. This approach allows assessment of particle size distribution and measurement reproducibility. (**B**) Morphological assessment of the purified VLPs with TEM. Representative TEM images were acquired at 10,000× magnification. Scale bar = 100 nm.

The characteristics of the four HPV L1 VLPs were analyzed using DLS and negative-stain TEM. In DLS, the size distribution of all particles was unimodal. Hydrodynamic diameters of HPV6, HPV11, and HPV18 VLPs were measured to be 59.1 nm, while that of HPV16 VLPs was 50.8 nm (Figure 5A). These results indicate that all four HPV serotypes exhibited similar hydrodynamic diameters of approximately 50–60 nm. Similarly, TEM analysis revealed that VLPs from all four HPV serotypes formed particles of approximately 50 nm in diameter, consistent with previously reported HPV VLPs (Figure 5B). Additionally, negative staining revealed the presence of internal cavities within the VLPs, indirectly confirming the absence of genetic material and the existence of empty spaces. In this study, the L1 proteins of four HPV serotypes (HPV 6, 11, 16, and 18) were successfully expressed using the baculovirus–insect cell hyper-expression system, and VLPs were subsequently assembled. All produced VLPs exhibited uniform particle size. 

### 3.5. Immunogenicity Analysis in Mice

**Figure 6:**
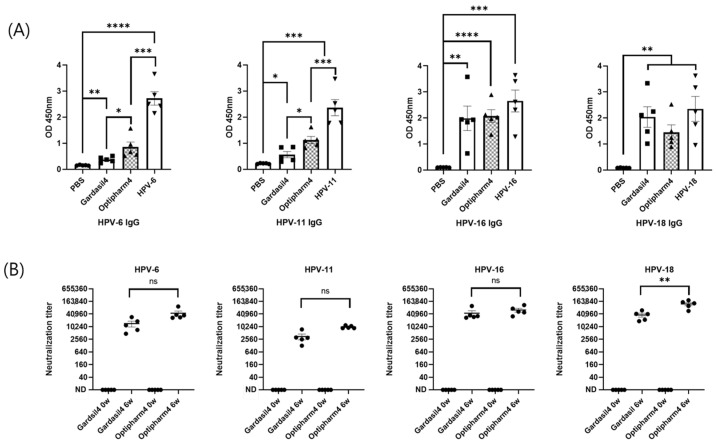
Humoral immune responses elicited by HPV VLPs in BALB/c mice. (**A**) Total anti-HPV IgG titers. (**B**) Neutralizing antibody titers against HPV types 6, 11, 16, and 18. Mice were immunized with purified HPV L1 VLPs (production and purification described in Figure 5). “0 W” indicates pre-immunization, and “6 W” indicates 6 weeks after the first immunization. Statistical significance was determined using an unpaired, two-tailed *t*-test in GraphPad Prism version 8.2.1. *p*-values were designated as follows: ns, not significant (*p* ≥ 0.05); * *p* < 0.05; ** *p* < 0.01; *** *p* < 0.001; **** *p* < 0.0001. IgG, Immunoglobulin G.

To evaluate the immunogenicity of HPV 6, 11, 16, and 18 VLPs produced using the hyper-expression vector system, groups of 6-week-old BALB/c mice were immunized intramuscularly with either the candidate VLPs or an equivalent dose of Gardasil as a positive control. The immunization comprised three intramuscular injections at 2-week intervals. The IgG titers of mouse antisera against the four HPV serotype VLPs, as measured using the IgG ELISA kit, exceeded the reference value of 10 units provided by the kit. Furthermore, the antibody titers were comparable to those induced by the quadrivalent Gardasil vaccine (Figure 6A). These results indicate that three immunizations with VLPs representing the four HPV serotypes elicited robust and high-titer antibody responses in mice. In this study, the neutralizing activity was evaluated using a PBNA and compared with that of the quadrivalent Gardasil vaccine. The results showed that the neutralizing antibody titers were equivalent to those of the Gardasil vaccine for three serotypes, while a significant difference was observed for HPV18 (Figure 6B).

## 4. Discussion

In this study, the baculovirus–insect cell hyper-expression system was successfully utilized to express L1 proteins of four HPV serotypes (HPV6, 11, 16, and 18) and assemble them into VLPs. The produced VLPs demonstrated a uniform size and elicited robust immune responses in mouse models, confirming their potential as effective vaccine candidates.

The structural characterization of the produced VLPs revealed morphologically uniform particles with diameters of 45–55 nm, consistent with authentic HPV capsid dimensions reported in previous studies. This structural fidelity is essential for maintaining the conformational epitopes required for neutralizing antibody induction. The self-assembly capacity of L1 proteins into VLPs in the absence of other viral components confirms the intrinsic property of L1 to form icosahedral capsids, as observed in other baculovirus-produced HPV VLP studies. The stability of these structures under various storage conditions suggests robust vaccine formulation potential, addressing one of the key requirements for commercial vaccine development.

Immunogenicity assessment in mouse models demonstrated that all four VLP types induced potent antibody responses comparable to those achieved by commercially available vaccines. These comprehensive immunogenicity findings, validated through the gold-standard PBNA methodology, confirm that baculovirus-produced HPV VLPs retain full antigenic integrity and immunogenic potential comparable to those of commercial vaccine formulations [33].

The first quadrivalent HPV vaccine (Gardasil) was licensed in 2006. Merck has held more than 80% of global sales, with Gardasil 9 alone. GSK discontinued Cervarix in many regions, further exacerbating supply constraints, and patent thickets around L1-VLP technology were cross-licensed between the two firms in 2005 [34]. Key Gardasil patents do not expire in major jurisdictions until 2028. The result is a quasi-monopoly in which list prices for the same biologic product range from 47 to 194 USD per dose in China [35] and about 190 USD in the United States, while the United Nations Children’s Fund pays only 4.55 USD under long-term supply agreements [36]. Although emerging HPV vaccine manufacturers, particularly from China, have entered the market, their broader participation is currently hindered by barriers, such as intellectual property rights, regulatory constraints, and limitations in funding. However, as demonstrated by the findings of this study and corroborated by previous research, it is reasonable to expect that the remaining technical challenges can be sufficiently overcome once these barriers are removed. Nevertheless, persistent supply shortages led the WHO to reduce its 2020 multi-age-cohort vaccination targets by half [37].

Currently, the global HPV vaccine market remains highly concentrated, with MSD’s Gardasil series dominating both production and distribution. Only three suppliers—MSD, GSK (Cervarix), and Innovax—hold WHO prequalification, sharply limiting procurement options for public sector programs. This quasi-monopolistic structure constrains competition, impedes price negotiation, and risks supply bottlenecks. Although several new candidates and manufacturers are in development, a meaningful expansion in market diversity has not yet materialized. To address these limitations and promote sustainability, diversification of vaccine production technologies and supplier base is essential. The alternative hyper-expression vector described in this study may represent one such avenue for broadening manufacturing capacity and enhancing market accessibility for HPV vaccines [38].

To date, extensive research has been conducted on HPV VLP vaccines, and currently, quadrivalent and nonvalent vaccines are widely used worldwide [39,40]. Although there have been various reports on HPV VLP production yields, the actual yields of commercial products remain speculative and are not precisely known [41]. The production yields of Gardasil and Cervarix have been estimated in previous studies to be approximately 29 mg/L and 40 mg/L, respectively. However, these values likely represent maximal estimates. In this study, we utilized a hyper-expression vector system and achieved average yields of approximately 67 mg/L, 51 mg/L, 25 mg/L, and 16 mg/L for the HPV 6, 11, 16, and 18 antigens, respectively, under laboratory-scale suspension culture conditions. This suggests that further increases in protein yield may be possible through process optimization, including scale-up, process control, and the application of fed-batch culture or bioreactor systems [42]. This represents the first example, other than reporter proteins, demonstrating that the use of a high-expression vector system for HPV VLP production can achieve higher yields than current commercial vaccines. These data confirm that technical innovation alone can reduce unit cost if downstream barriers are addressed.

## 5. Conclusions

This study successfully demonstrated the application of a hyper-expression vector system for producing HPV VLPs and evaluated their potential for large-scale industrial manufacturing. These results are consistent with previous studies using high-expression vectors with EGFP, demonstrating enhanced expression and yield of the target protein. Therefore, this study further supports the commercial potential of high-expression vectors for large-scale protein production. The HPV VLPs representing four serotypes (6, 11, 16, and 18) exhibited uniform morphological characteristics and demonstrated immunogenicity profiles comparable to those of existing commercial vaccines. These findings suggest that the hyper-expression system maintains the antigenic properties essential for vaccine efficacy while achieving enhanced productivity with substantial commercial advantages. The improved manufacturing efficiency demonstrated by this approach indicates its potential to enable the development of cost-effective HPV vaccines, which could contribute to addressing critical market access challenges driven by current monopolistic pricing structures. Future research directions should focus on expanding the application of hyper-expression vector technology to develop a comprehensive portfolio of cost-effective vaccine manufacturing solutions. Specifically, investigations will explore the extension beyond the current 4-valent formulation to encompass 9-valent or 12-valent HPV serotype coverage using novel hyper-expression vector platforms. The development of such competitive manufacturing platforms represents not only a commercial opportunity but also a critical public health imperative for achieving the WHO’s global strategy for cervical cancer elimination.

## Data Availability

All data used during the study are available from the corresponding author upon request.

## Supplementary Materials

The following supporting information can be downloaded at: https://www.mdpi.com/article/10.3390/vaccines13101006/s1, Sequence S1: HPV L1 codon optimization sequence.

## Abbreviations

The following abbreviations are used in this manuscript:

VLP	Virus-like particle
HPV	Human papillomavirus
TEM	Transmission electron microscopy
DLS	Dynamic light scattering
WHO	World Health Organization
EGFP	Enhanced Green Fluorescent Protein
MOI	Multiplicity of infection
PBS	Phosphate-buffered saline
SDS-PAGE	Sodium dodecyl sulfate polyacrylamide gel electrophoresis
HRP	Horseradish peroxidase
IgG	Immunoglobulin G
ELISA	Enzyme-linked immunosorbent assay
BME	β-mercaptoethanol
PCR PBNA	Polymerase chain reaction pseudovirus-based neutralization assay
IACUC	Institutional Animal Care and Use Committee
