# Supplementary material for: Productivity Improvement of Human Papillomavirus-like Particles in Insect Cells Using Hyper-Expression Baculovirus Vector
Source: Vaccines (Basel). 2025 Sep 25;13(10):1006. doi: 10.3390/vaccines13101006 (PMC12568247; doi:10.3390/vaccines13101006)
Supplement: Supplementary file 1 [file vaccines-13-01006-s001.zip › vaccines-3844720-supplementary.pdf]

Sequence S1: HPV L1 codon optimization sequence

>HPV6-L1-codon optimization

ATGTGGCGCCCTAGCGACAGCACCGTATACGTGCCTCCTCCTAACCCTGTCTCCAAGGTTGTTGCCACGGACG
CTTATGTTACCCGCACCAACATCTTCTACCACGCCTCGAGTTCTAGACTCCTTGCAGTCGGACATCCTTACTTTT
CCATTAAGAGGGCTAATAAAACTGTTGTACCAAAAGTGTCAGGCTACCAGTACAGAGTCTTCAAAGTGGTGTT
ACCAGATCCAAACAAGTTTGCTCTGCCTGACTCCTCTTTGTTCGATCCAACGACACAACGTCTGGTGTGGGCAT
GCACTGGTCTAGAGGTCGGGAGGGGCCAACCATTGGGCGTGGGTGTATCAGGACACCCTTTCCTGAATAAGT
ACGACGATGTTGAAAACTCAGGTAGTGGTGGAAACCCTGGACAGGACAACCGTGTCAACGTCGGCATGGATT
ATAAGCAAACACAGCTCTGCATGGTGGGATGTGCGCCGCCTTTGGGCGAGCACTGGGGTAAGGGTAAACAAT
GTACTAATACACCTGTACAGGCCGGTGACTGCCCGCCCTTGGAGCTCATCACCAGTGTAATCCAGGATGGTGAT
ATGGTTGACACCGGCTTCGGTGCTATGAACTTCGCTGACCTCCAGACGAATAAATCAGACGTGCCTATCGACAT
ATGTGGCACGACATGCAAATATCCAGACTACCTGCAGATGGCGGCAGATCCATACGGTGATCGTTTGTTCTTCT
TCCTGCGTAAGGAACAGATGTTCGCCAGACATTTCTTTAACAGGGCTGGAGAAGTGGGGGAGCCAGTGCCTG
ACACTCTTATCATTAAGGGTAGCGGAAACCGCACGTCGGTAGGCTCTTCGATCTATGTCAACACCCCCAGCGG
CTCACTGGTGTCCAGCGAAGCTCAACTCTTTAACAAGCCTTACTGGCTACAAAAGGCCCAGGGTCACAACAA
TGGAATTTGCTGGGGAAATCAACTGTTCGTTACTGTCGTCGATACTACACGTAGTACCAACATGACATTATGTGC
CTCCGTCACTACATCTTCCACCTACACCAACTCAGACTACAAGGAGTACATGCGCCACGTGGAAGAATACGAT
CTGCAGTTCATCTTCCAGCTGTGCAGCATCACCCTAAGCGCGGAGGTGATGGCATACATTCATACAATGAATCC
TTCTGTTCTTGAGGACTGGAACTTCGGCTTATCGCCGCCACCAAACGGTACGCTCGAAGACACCTACAGATAC
GTGCAATCACAGGCCATCACCTGTCAAAAGCCCACTCCCGAAAAGGAGAAACCAGACCCCTACAAGAACCT
GTCGTTTTGGGAGGTAAATCTGAAGGAGAAGTTCTCTAGTGAACTCGATCAGTATCCTTTGGGACGCAAATTCT
TGTTGCAATAA

>HPV11-L1-codon optimization

ATGAGCGACAGCACTGTGTACGTGCCCCCTCCCAACCCTGTATCCAAGGTTGTAGCCACGGACGCCTACGTGA
AACGCACCAACATCTTCTACCACGCTTCGAGTTCAAGACTGCTTGCTGTGGGACACCCATACTACTCTATTAAG
AAGGTGAACAAGACTGTTGTACCAAAGGTATCCGGGTACCAGTATAGAGTCTTCAAAGTGGTTTTGCCAGATC
CGAACAAGTTTGCACTGCCGGATTCATCTCTGTTCGACCCCACTACACAGCGTCTGGTATGGGCGTGCACAGG
ATTGGAGGTGGGCAGGGGTCAACCTCTCGGAGTCGGTGTGTCGGGACACCCATTGCTCAACAAATATGACGA
TGTTGAGAATAGTGGTGGATATGGTGGCAATCCCGGCCAGGACAACCGTGTCAACGTTGGTATGGATTACAAA
CAAACCCAGCTATGTATGGTCGGCTGTGCTCCTCCTTTAGGTGAACATTGGGGTAAGGGAACGCAATGCTCTA
ACACCTCCGTCCAAAATGGCGATTGCCCCCCGTTGGAGCTTATCACCTCCGTGATACAAGATGGCGACATGGTT
GATACCGGATTCGGTGCCATGAACTTCGCCGACTTACAGACCAATAAGTCGGACGTTCCCCTGGACATCTGTG
GAACTGTCTGCAAATACCCCGACTATTTGCAAATGGCGGCAGACCCTTATGGTGATAGGCTCTTCTTCTACTTG

CGCAAGGAGCAGATGTTTGCACGTCACTTCTTCAACCGCGCCGGAACGGTAGGGGAACCTGTGCCAGACGA
TCTGTTGGTCAAGGGAGGCAATAATAGATCATCTGTCGCTAGTAGTATCTATGTCCATACACCTAGTGGCTCACT
CGTGTCGAGCGAGGCTCAACTCTTTAACAAGCCATACTGGCTGCAGAAGGCTCAGGGCCACAACAACGGTAT
TTGCTGGGGTAACCACCTGTTCGTTACTGTGGTAGACACCACTCGTAGCACAAATATGACACTATGTGCGTCCG
TGTCTAAATCCGCTACTTACACGAACTCTGACTACAAAGAATACATGCGCCACGTGGAGGAGTTCGACCTGCA
GTTCATCTTCCAACTCTGTAGCATTACTCTTTCCGCAGAAGTCATGGCTTATATCCATACCATGAACCCTAGTGTC
TTGGAAGATTGGAACTTCGGTTTATCGCCGCCCCCTAATGGTACACTGGAAGATACTTACAGATACGTACAGTC
ACAGGCGATCACCTGCCAGAAACCAACGCCTGAGAAGGAGAAGCAGGACCCCTACAAGGACATGTCCTTCT
GGGAGGTTAACCTCAAAGAGAAGTTTTCCTCTGAACTAGACCAGTTTCCACTGGGACGTAAATTCCTGTTGCA
ATCAGGATACAGGGGCCGCACTTCTGCCAGGACGGGCATCAAGCGACCAGCTGTGAGCAAGCCCTCTTAA

>HPV16-L1-codon optimization

ATGTCTCTGTGGCTGCCTAGTGAGGCCACCGTGTACCTGCCTCCTGTCCCAGTGTCCAAGGTCGTGAGCACCGA
TGAATACGTGGCTCGCACCAACATCTACTACCACGCTGGAACTTCCAGACTGCTGGCTGTGGGACACCCCTAC
TTCCCCATCAAGAAGCCTAACAACAACAAGATCCTCGTGCCTAAGGTGAGCGGTCTGCAATACAGGGTGTTCC
GTATCTACCTCCCCGACCCCAACAAGTTCGGATTCCCTGATACCTCATTCTACAACCCAGACACACAGCGCCTG
GTCTGGGCTTGCGTCGGTGTGGAAGTGGGTCGTGGACAGCCACTCGGTGTGGGCATCAGTGGCCACCCTCTG
CTGAACAAGTTGGATGACACAGAGAACGCTAGTGCCTACGCTGCTAACGCTGGCGTGGATAACCGCGAGTGC
ATCTCTATGGACTACAAGCAAACCCAGTTGTGCCTGATCGGTTGCAAGCCCCCTATCGGTGAACACTGGGGCA
AGGGATCCCCTTGCAACAACGTGGCTGTGACACCAGGTGACTGCCCCCCACTGGAACTGATCAACACCGTGA
TCCAAGATGGCGACATGGTGGATACCGGCTTCGGTGCTATGGACTTCACCACACTGCAGGCTAACAAGAGTG
AAGTGCCTCTGGATATCTGCACATCTATCTGCAAGTACCCTGACTACATCAAGATGGTGTCAGAGCCTTACGGC
GACAGCCTGTTCTTCTACCTGCGTAGGGAGCAAATGTTCGTCAGACACCTGTTCAACCGCGCTGGCGCTGTGG
GAGAAAACGTGCCAGACGACCTGTACATCAAGGGCTCCGGTTCTACTGCTAACCTGGCCAGTTCAAACTACTT
CCCTACACCTAGTGGTTCTATGGTGACCTCCGATGCCCAAATCTTCAACAAGCCTTACTGGCTGCAAAGAGCTC
AGGGCCACAACAACGGCATCTGCTGGGGTAACCAGCTGTTCGTGACTGTGGTGGACACTACCCGCTCAACCA
ACATGAGCCTGTGCGCTGCCATCTCCACTTCAGAACCTACCTACAAGAACACCAACTTCAAGGAGTACCTGAG
GCACGGTGAGGAATACGACCTGCAGTTCATCTTCCAGCTGTGCAAGATCACCCTCACTGCCGACGTGATGAGC
TACATCCACTCCATGAACTCCACTATCTTGGAGGACTGGAACTTCGGTCTCCAACCACCTCCAGGAGGCACCT
TGGAGGATACCTACCGTTTCGTCACAAGCCAGGCTATCGCTTGCCAGAAGCACACACCTCCAGCTCCCAAGG
AAGATCCCTTGAAGAAGTACACTTTCTGGGAAGTGAACCTGAAGGAAAAGTTCTCTGCCGACTTGGATCAATT
CCCCCTGGGAAGAAAGTTCCTGCTGCAATAA

>HPV18-L1-codon optimization

ATGGCTTTGTGGCGCCCTTCCGACAACACCGTCTACCTGCCACCTCCTTCTGTCGCTAGAGTGGTGAACACAG
ATGATTACGTGACTCGCACAAGCATCTTCTACCACGCTGGCAGCTCTCGCCTCCTGACTGTGGGTAACCCATAC
TTCAGGGTCCCTGCTGGTGGTGGCAACAAGCAGGATATCCCTAAGGTGTCCGCCTACCAATACAGAGTGTTCA
GGGTGCAGCTGCCTGACCCAAACAAGTTCGGTCTGCCTGATACTAGTATCTACAACCTGGAGACCCAGCGTCT
CGTGTGGGCCTGCGCTGGAGTGGAAATCGGCCGTGGTCAGCCCCTGGGTGTGGGCCTGAGTGGTCACCCTTT
CTACAACAAGCTGGATGACACTGAAAGTTCCCACGCCGCCACCTCCAACGTCTCTGAGGACGTGAGGGACAA
CGTGTCTGTGGATTACAAGCAGACACAGCTGTGCATCTTGGGCTGCGCCCCTGCTATCGGAGAGCACTGGGCT
AAGGGCACCGCTTGTAAGTCCCGTCCTCTGTCCCAGGGCGACTGCCCCCCTCTCGAACTGAAGAACACCGTGT
TGGAAGATGGTGATATGGTGGACACTGGATACGGTGCCATGGACTTCTCCACCTTGCAAGACACCAAGTGTGA
GGTGCCACTCGACATCTGCCAGTCTATCTGCAAGTACCCTGATTACTTGCAAATGTCAGCTGATCCTTACGGTG
ATTCCATGTTCTTCTGCCTGCGCCGTGAGCAACTGTTCGCCCGCCACTTCTGGAACAGGGCTGGCACCATGGG
TGATACTGTGCCTCAATCACTGTACATCAAGGGCACAGGTATGCGTGCTTCACCCGGCAGCTGTGTGTACTCCC
CCAGCCCATCCGGCTCTATCGTGACCTCTGACTCCCAATTGTTCAACAAGCCATACTGGCTGCACAAGGCTCA
AGGTCACAACAACGGTGTCTGCTGGCACAACCAACTGTTCGTGACTGTGGTGGATACCACCCGCAGTACCAA
CCTGACAATCTGTGCTTCTACTCAGTCCCCCGTGCCTGGTCAGTACGACGCTACCAAGTTCAAGCAGTACAGCC
GCCACGTGGAGGAATACGACTTGCAGTTCATCTTCCAATTGTGTACTATCACTCTGACCGCTGATGTCATGTCCT
ACATCCACTCAATGAACAGCAGTATCCTGGAGGATTGGAACTTCGGTGTGCCCCCCCCTCCAACCACTAGTTT
GGTGGACACATACCGCTTCGTGCAATCTGTGGCTATCACCTGTCAGAAGGATGCTGCTCCTGCTGAGAACAAG
GACCCCTACGACAAGCTCAAGTTCTGGAACGTGGACCTGAAGGAGAAGTTCTCTCTCGACCTGGATCAATACC
CCCTGGGAAGGAAGTTCTTGGTGCAGTAA
